# Self‐Powered Artificial Mechanoreceptor Based on Triboelectrification for a Neuromorphic Tactile System

**DOI:** 10.1002/advs.202105076

**Published:** 2022-01-14

**Authors:** Joon‐Kyu Han, Il‐Woong Tcho, Seung‐Bae Jeon, Ji‐Man Yu, Weon‐Guk Kim, Yang‐Kyu Choi

**Affiliations:** ^1^ School of Electrical Engineering Korea Advanced Institute of Science and Technology (KAIST) 291 Daehak‐ro, Yuseong‐gu Daejeon 34141 Republic of Korea; ^2^ Electronics Engineering Department Hanbat National University 125 Dongseo‐daero, Yuseong‐gu Daejeon 34158 Republic of Korea

**Keywords:** biristor neuron, breath monitoring, mechanoreceptors, spiking neural network, triboelectric nanogenerators

## Abstract

A self‐powered artificial mechanoreceptor module is demonstrated with a triboelectric nanogenerator (TENG) as a pressure sensor with sustainable energy harvesting and a biristor as a neuron. By mimicking a biological mechanoreceptor, it simultaneously detects the pressure and encodes spike signals to act as an input neuron of a spiking neural network (SNN). A self‐powered neuromorphic tactile system composed of artificial mechanoreceptor modules with an energy harvester can greatly reduce the power consumption compared to the conventional tactile system based on von Neumann computing, as the artificial mechanoreceptor module itself does not demand an external energy source and information is transmitted with spikes in a SNN. In addition, the system can detect low pressures near 3 kPa due to the high output range of the TENG. It therefore can be advantageously applied to robotics, prosthetics, and medical and healthcare devices, which demand low energy consumption and low‐pressure detection levels. For practical applications of the neuromorphic tactile system, classification of handwritten digits is demonstrated with a software‐based simulation. Furthermore, a fully hardware‐based breath‐monitoring system is implemented using artificial mechanoreceptor modules capable of detecting wind pressure of exhalation in the case of pulmonary respiration and bending pressure in the case of abdominal breathing.

## Graphical abstract

A self‐powered artificial mechanoreceptor module is demonstrated for a neuromorphic tactile system. It can greatly reduce the required amount of energy compared to the conventional tactile system based on a von Neumann computer. It can detect low pressures near 3 kPa due to the high output range of the triboelectric nanogenerator. A fully hardware‐based breath‐monitoring system is implemented.

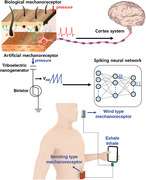

## 1 Introduction

Artificial tactile perception systems are useful in the development of robotics, prosthetics, and medical and healthcare devices because they are capable of object, pattern, or texture recognition with high accuracy by processing the signals received from sensor arrays through a deep neural network (DNN).^[^ ^1^, ^2^, ^3^, ^4^ ^]^ However, most of these systems are based on software that requires an advanced von Neumann computer, which is difficult to integrate into edge devices owing to its high power requirements. Implementation of an artificial tactile perception system on edge devices such as, an IoT sensor, a smartphone, and a smart watch is important because communication failure between edge devices or between an edge device and a server computer can cause significant economic loss and human injury.^[^ ^5^, ^6^, ^7^ ^]^ If the power consumed by edge devices is high due to the use of a von Neumann computer, energy should be repeatedly supplied, which can be difficult to manage by periodically replacing or recharging a battery. Therefore, a different sustainable approach should be developed as opposed to a bulky computer consuming a large amount of power in order to implement a scalable artificial tactile perception system on edge devices.

**Figure 1:**
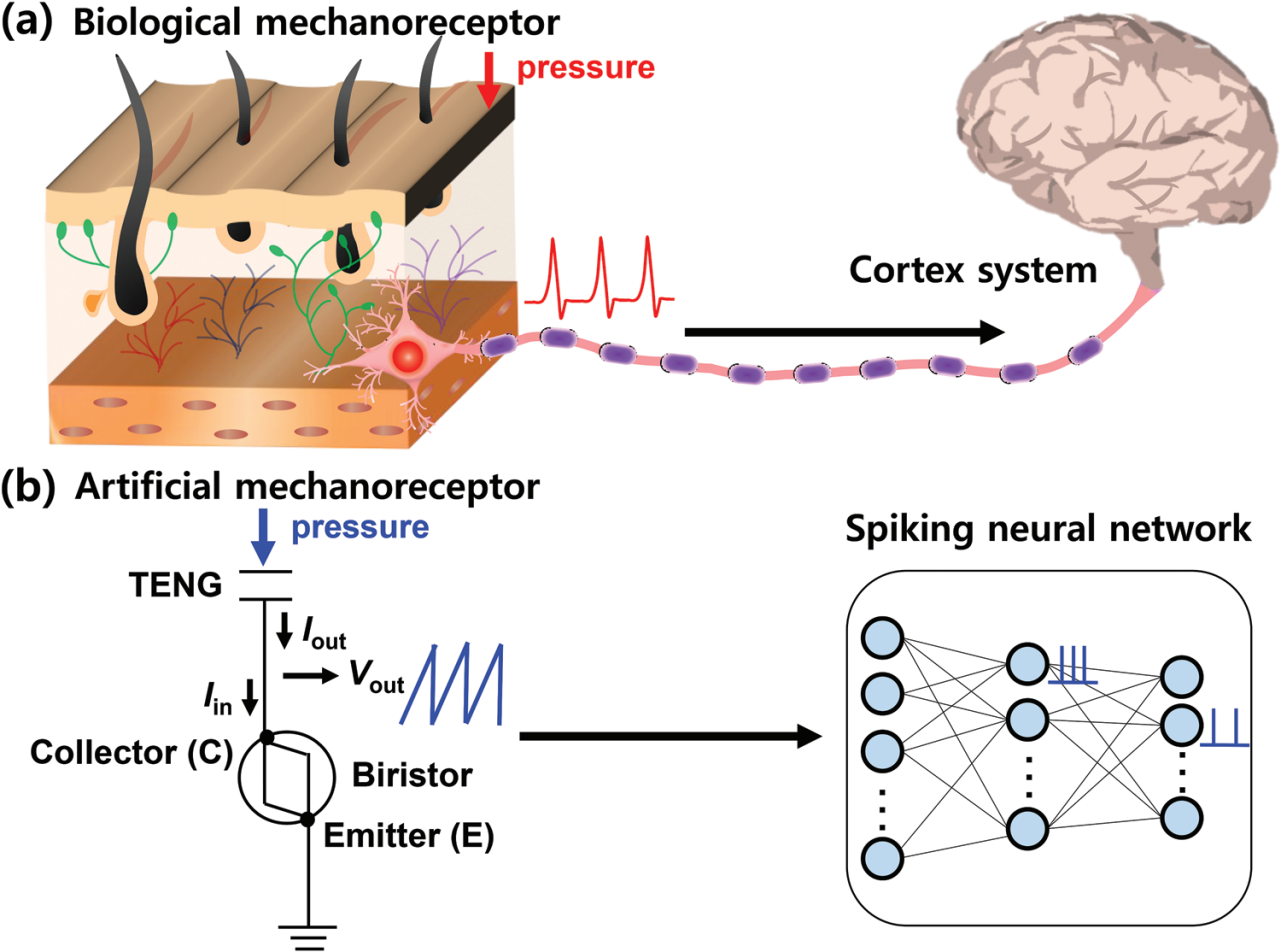
Biological and artificial tactile perception system: a) Schematic of the biological tactile perception system. Mechanoreceptors generate spike signals when pressure is detected and send these signals to the cortex for neural processing. The spikes are generated more frequently when the intensity of the stimuli increases. b) Schematic of the artificial tactile perception system. The self‐powered artificial mechanoreceptor module consists of a triboelectric nanogenerator (TENG) sensor with energy harvesting and a biristor neuron; it can generate spike signals that vary depending on the applied pressure. The spike signals are transmitted to the synapses and neurons that compose the spiking neural network (SNN) for neural processing.

A biological tactile perception system recognizes objects, patterns, or textures by transmitting sensory information in the form of spikes, and is considered a key approach to realize low energy consumption.^[^ ^8^, ^9^, ^10^, ^11^, ^12^, ^13^ ^]^ The imitation of biological spike transmission is considered a powerful means to build a low‐power tactile perception system, and neuromorphic tactile perception systems based on spiking neural networks (SNNs) have thus attracted considerable attention.^[^ ^14^, ^15^, ^16^, ^17^ ^]^ In addition, von Neumann bottlenecks between the central processing unit and the memory for the implementation of software‐based DNNs can also be eliminated. Therefore, neuromorphic tactile perception systems based on SNNs are advantageous when applied to edge devices given their low power consumption. The first step toward neuromorphic tactile perception is to convert the pressure signals into spikes so that they can be used as inputs to SNNs because signals acquired from the environment are in the continuous and analog domain. In biology, mechanoreceptors perform this function, accepting mechanical signals and transmitting electrical spike signals to the cortex for neural processing, as illustrated in **Figure** **1**a.^[^ ^10^, ^11^, ^12^ ^]^ However, general pressure sensors cannot be applied because the outputs are not in the form of spikes.

In relation to this, Zhang et al. demonstrated an artificial mechanoreceptor module with a piezoelectric nanogenerator and a Mott memristor neuron.^[^ ^14^ ^]^ It could generate spikes as a function of the input pressure, which is a desirable characteristic of an artificial mechanoreceptor. The module did not require an extra power supply to generate spikes due to its use of a nanogenerator. Although this work set a milestone in self‐powered artificial sensory systems, further studies should be performed to use them to practical applications such as, robotics, prosthetics, medical, or healthcare devices, because the response to low pressure levels is important for these applications, considering that human can feel gentle touches during daily activities less than 10 kPa.^[^ ^18^, ^19^, ^20^ ^]^ Recently, Li et al. demonstrated an artificial mechanoreceptor module capable of responding to pressures of less than 10 kPa, but an additional power source was necessary because the pressure sensor was a type of resistor.^[^ ^21^ ^]^ Another approach that is capable of detecting low pressure levels without a power source therefore should be developed. In addition, further system‐level studies should be performed to utilize an artificial mechanoreceptor in an actual tactile perception system.

In this work, a self‐powered artificial mechanoreceptor module composed of a triboelectric nanogenerator (TENG) for a sensor with sustainable energy and a biristor for a neuron was demonstrated, not as a complex circuit but rather as a single device. The name of the biristor stems from a bi‐stable resistor that can stably make a high‐resistance state (HRS) and a low‐resistance state (LRS), and serve as a single transistor neuron by directly converting input current to output voltage spiking.^[^ ^22^ ^]^ When pressure is applied, the output current from the TENG is transmitted to the biristor neuron as an input current. Thereby the characteristic of a mechanoreceptor is realized, where the spiking varies with the applied pressure. Unlike previous work, it can detect low pressures near 3 kPa because the output range of the TENG is much higher than that of other types of nanogenerators.^[^ ^23^, ^24^ ^]^ It should be noted that the TENG is considered a promising candidate for application to an artificial tactile perception system, as it offers the advantages of high output, flexibility, and light weight.^[^ ^25^, ^26^, ^27^, ^28^, ^29^ ^]^ The biristor neuron is considered a possible candidate to replace bulky and high‐energy‐consuming CMOS circuit‐based spiking neurons for SNNs.^[^ ^30^, ^31^, ^32^ ^]^ After analyzing the characteristics of the TENG and the biristor neuron, a self‐powered artificial mechanoreceptor module capable of detection near 3 kPa that is able to sense a gentle touch such as, handwriting and breathing is realized by connecting the two abovementioned components. Based on the spiking characteristics according to the pressure, classification of handwritten digits in the Modified National Institute of Standards and Technology (MNIST) dataset is performed with the aid of a software simulation in order to demonstrate that complex pattern recognition is possible with the neuromorphic tactile system composed of an artificial mechanoreceptor module. Furthermore, a fully hardware‐based breath‐monitoring system that can classify exhalation and inhalation is implemented by using wind pressure of exhalation in the case of pulmonary respiration and bending pressure in the case of abdominal breathing. The proposed multi‐way detectability of the artificial mechanoreceptor module that can sense different types of pressure is favorable for various healthcare applications.

## 2 Results and Discussion

### 2.1 Structure of an Artificial Mechanoreceptor Module

A self‐powered artificial mechanoreceptor module was implemented by serially connecting a TENG sensor with energy harvesting and a biristor neuron, as shown in Figure 1b. When pressure is applied to the TENG, the generated current is transmitted to the collector (C) of the biristor neuron and spike‐shaped voltage signals are generated from the same C of the biristor neuron. Therefore, a set consisting of a TENG sensor and a biristor neuron can act as an artificial mechanoreceptor module that generates spike signals when pressure is detected. In addition, as the applied pressure to the TENG increases, the spiking frequency (*f*) of the artificial mechanoreceptor module increases because a greater level of current is transmitted to the biristor neuron from the TENG. Note that an extra power source is unnecessary to implement the artificial mechanoreceptor, which is advantageous for a low‐power tactile perception system. Hybrid integration to combine a TENG and an electronic device such as a transistor and a nonvolatile memory becomes increasingly important for self‐powered electronics.^[^ ^33^, ^34^ ^]^ The proposed TENG is like a self‐aware sensor that can awaken when an external stimulus is applied. The spike signals will be further transmitted to the next synapses and neurons that compose the SNN.

### 2.2 Characteristics of a Triboelectric Nanogenerator

**Figure 2:**
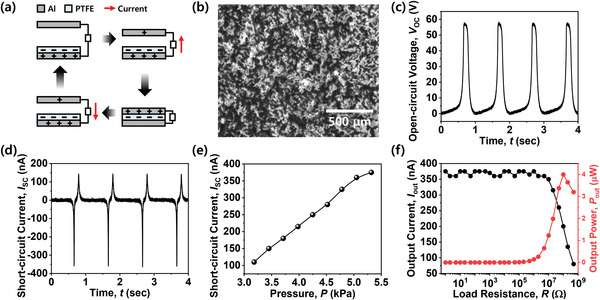
Characteristics of the TENG: a) Schematic of the operational mechanism of the fabricated TENG. b) Scanning electron microscopy (SEM) image of the surface of the PTFE film. A micro‐nano morphology was formed on the surface of the PTFE film. c) Open‐circuit voltage (*V*_OC_) of the TENG with a pressure level of 5.3 kPa. d) Short‐circuit current (*I*_SC_) of the TENG with a pressure level of 5.3 kPa. e) Short‐circuit current (*I*_SC_) according to the pressure of the TENG. *I*_SC_ increased when more pressure was applied. f) Output current (*I*_out_) and output power (*P*_out_) according to the load resistance (*R*) of the TENG.

In this work, a TENG comprising two aluminum (Al) plates as an electrode and a polytetrafluoroethylene (PTFE) film as a triboelectric layer was utilized, as shown in **Figure** **2**a. It is well known that a TENG is advantageous for adapting its structure according to various mechanical input stimuli with a variety of forms customized to a targeted device. The fabrication details are provided in Figure S1, Supporting Information. The TENG has a length and a width of 4 cm, and the thickness of the PTFE film is 100 µm. In addition, a micro‐nano hierarchical morphological structure was formed on the surface of the PTFE film in order to increase the electrical output and improve the pressure sensitivity by enlarging the effective contact area during the electrification via contact‐separation between the Al electrode and the PTFE. For the formation of the micro‐nano hierarchical morphology, gold nano‐islands, which were served as hard mask during subsequent etching, were evaporated on the surface of the PTFE film. Upon subsequent plasma etching with a mixed gas of O_2_, Ar, and CF_4_, a vertical rod‐like micro‐nano morphology was formed on the surface of the PTFE film, as shown in Figure 2b. This TENG is operated by linear force in contact‐separation mode. Alternating current (AC) power is generated by alternating contact and separation of the upper electrode and the PTFE film. The lower electrode is attached onto the rear surface of the PTFE film. The detailed operational mechanism of this TENG is depicted in Figure 2a. The surface of the PTFE film is charged negatively via contact electrification with the upper electrode. When the upper electrode is forced to get close to the PTFE film, current flows from the lower electrode to the upper electrode. Subsequently, when the upper electrode moves away from the PTFE film, current flows from the upper electrode to the lower electrode. As the contact pressure increases, the magnitude of the current of the TENG increases due to a greater amount of surface charge.^[^ ^35^ ^]^ With the micro‐nano morphology of the PTFE film, high pressure sensitivity can be achieved.^[^ ^36^ ^]^ The measured open‐circuit voltage (*V*_OC_) and short‐circuit current (*I*_SC_) of the TENG were ≈60 V and 370 nA, respectively, with a contact pressure of 5.3 kPa, as correspondingly shown in Figure 2c,d. When the contact pressure was increased from 3.2 kPa, the *I*_SC_ of the TENG increased linearly, as presented in Figure 2e. To investigate the output power (*P*_out_) of the TENG, the output current (*I*_out_) was measured when the load resistance (*R*) ranged from 1 Ω to 500 MΩ, as shown in Figure 2f. As *R* increased from 10 MΩ, *I*_out_ decreased. In addition, *P*_out_ was calculated using the following equation:

(1) $$P_{\mathrm{out}}={I_{\mathrm{out}}}^{2}\;R$$

A maximum instantaneous *P*_out_ of 4 µW was achieved with an *R* value of 100 MΩ. The output characteristics of this TENG are high even with a small amount of contact pressure. This is attributed to two features. One is that the PTFE is a highly triboelectric negative material and the other is that the effective contact area is enhanced by the micro‐nano morphology.

### 2.3 Characteristics of a Biristor Neuron

**Figure 3:**
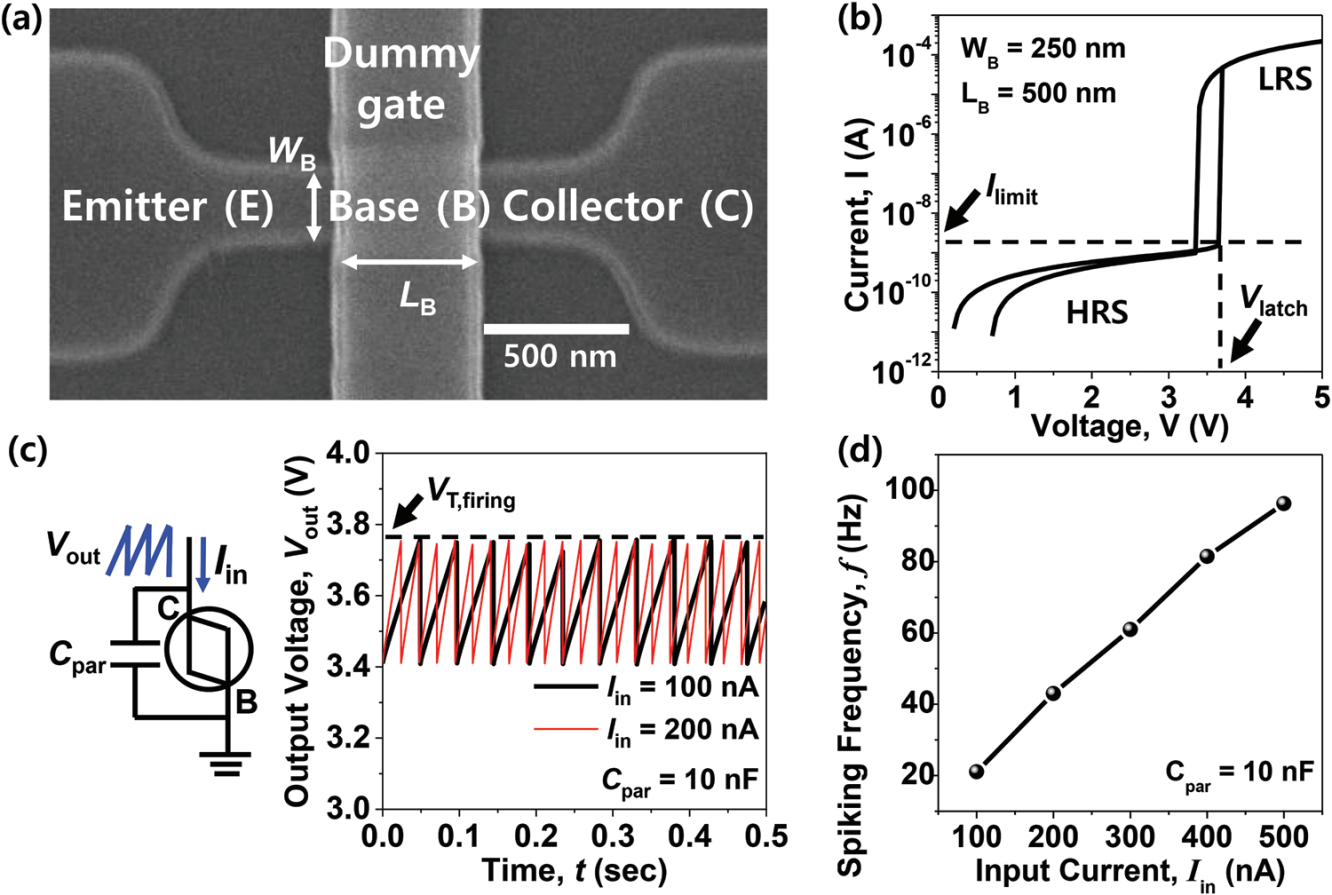
Characteristics of the biristor neuron: a) SEM image of the fabricated biristor neuron. b) Current–voltage characteristics (*I*–*V*) of the fabricated biristor neuron. A large amount of current abruptly flowed at a certain latch‐up voltage (*V*_latch_) by the single‐transistor latch (STL) phenomenon, which allows the firing step during the operation of the neuron. c) Spiking characteristics (*V*_out_–*t*) of the fabricated biristor neuron, showing the typical spiking properties of a integrate‐and‐fire (IF) neuron. d) Spiking frequency (*f*) according to the input current (*I*_in_) of the fabricated biristor neuron. Here, *f* increased with an increase in *I*_in_ because the integration speed increased.

A biristor neuron can mimic a neuronal integrate‐and‐fire (IF) function and spiking operation by means of the single‐transistor latch (STL) phenomenon.^[^ ^30^, ^31^, ^32^ ^]^ In this work, a biristor neuron with a base width (*W*_B_) of 250 nm and a base length (*L*_B_) of 500 nm was fabricated, as shown in the scanning electron microscope (SEM) image in **Figure** **3**a. The fabrication details are provided in Figure S2, Supporting Information. Starting with p‐type (100) silicon‐on‐insulator (SOI) wafer, an active area was patterned using photo‐lithography and plasma etching. Afterwards, a dummy gate with gate dielectrics was deposited and patterned by another photo‐lithography and plasma etching. Then, doping for an emitter and a collector (E/C) was followed with arsenic implantation and the E/C dopants were activated by subsequent rapid thermal annealing (RTA). After that, base (B) doping was performed by boron implantation and subsequent RTA was followed. Figure 3b shows the current–voltage (*I*–*V*) characteristics of the fabricated biristor neuron, which was measured with a parameter analyzer. Note that a large current abruptly flowed at a certain latch‐up voltage (*V*_latch_) via the STL phenomenon. In detail, increased voltage makes electrons in the E be injected to the B, which trigger impact ionization and *e‐h* pair generation. Then, the generated holes are stored in the B and reduce the potential barrier between the E and the B. Therefore, more electrons are injected into the B and more proliferated holes are generated by the iterative impact ionization. This is a kind of positive feedback that triggers latch‐up, which is a catastrophic current change. As a result, the device was in a HRS when the applied voltage was lower than *V*_latch_, changing to a LRS when the applied voltage exceeded *V*_latch_. This allows the instant firing and spiking operation of the neuron.^[^ ^30^, ^31^, ^32^ ^]^

To confirm the neuronal operation, constant input current (*I*_in_) that corresponds to the output current (*I*_out_) from the aforementioned TENG was applied to the C and the output voltage (*V*_out_) was measured at the same C while the E was grounded. An external parasitic capacitor with capacitance (*C*_par_) of 10 nF was connected to the biristor in parallel. Figure 3c shows the neuronal spiking characteristics of the biristor neuron, which is represented by *V*_out_ according to time. When *I*_in_ was applied to C, positive charges were integrated because the device was initially in the HRS. According to this integration process of the IF neuron, *V*_out_ measured at C was increased by the integration of the positive charges in the parasitic capacitor. When *V*_out_ reached the firing threshold voltage (*V*_T,firing_), which is identical to *V*_latch_, the integrated charges in the parasitic capacitor were suddenly fired because the biristor changed to the LRS via the STL. In this way, neuronal spiking characteristics could be achieved by iterative integration and firing when a constant *I*_in_ was applied. As shown in Figure 3d, *f* increased when *I*_in_ increased because the integration speed was increased, which can be represented by the following equation: $=\frac{I_{\mathrm{in}}}{C_{\mathrm{par}}\times \left(V_{T,\mathrm{firing}}-V_{\mathrm{low}}\right)}\;$, where *V*_low_ is the bottom output voltage of the spiking process. Therefore, it can be expected that *f* of the artificial mechanoreceptor module increases when the *I*_out_ from the TENG increases due to the higher pressure. It is worth noting that *I*_in_ should be higher than the lower boundary of the forbidden region (*I*_limit_) marked in Figure 3b.^[^ ^30^, ^31^, ^32^ ^]^ Unless *I*_in_ is larger than *I*_limit_, charge integration cannot be enabled. This feature is similar to a biological neuron function where spiking is disabled when the input stimulus does not exceed a threshold value.

### 2.4 Characteristics of an Artificial Mechanoreceptor Module

**Figure 4:**
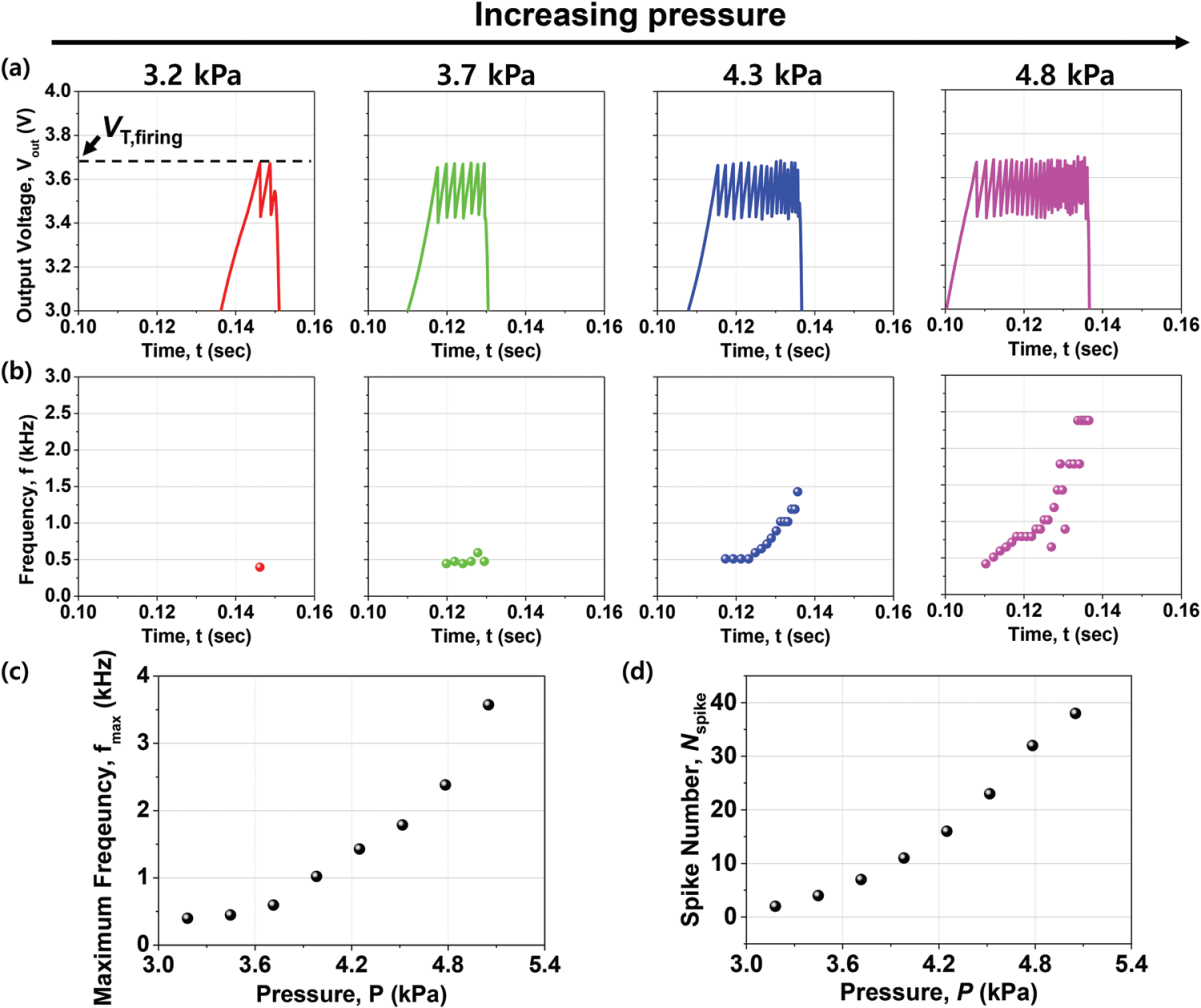
Characteristics of the artificial mechanoreceptor module: a) Spiking characteristics (*V*_out_–*t*) of the artificial mechanoreceptor module with various applied pressures. b) Spiking frequency (*f*) over time extracted from *V*_out_–*t*. c) Maximum frequency (*f*_max_) as a function of the pressure (*P*). d) The number of spikes during one touch (*N*_spike_) as a function of *P*. Here, *f*_max_ and *N*_spike_ increased as *P* increased, which is a desired characteristic of the mechanoreceptor.

**Figure** **4** shows the spiking characteristics of the artificial mechanoreceptor module obtained by connecting the TENG sensor and the biristor neuron. Detailed configuration of the electrical connection between them is shown in Figure S3, Supporting Information. Pressure was applied with an electrodynamic shaker and *V*_out_ was measured using a parameter analyzer. As shown in Figure 4a, when the pressure was applied, *V*_out_ increased until it reached *V*_T,firing_ because the *I*_out_ from the TENG flowed into the biristor neuron as the *I*_in_. Spiking was then repeated until the Al electrode and the PTFE were separated from each other. *V*_out_ decreased rapidly when they were far apart because the output current from the TENG flowed out in a direction opposite to that of the biristor neuron. Figure 4b shows *f* of each spike extracted from Figure 4a, and Figure 4c shows the maximum frequency (*f*_max_) as a function of the pressure. It was confirmed that *f*_max_ increased as the pressure increased because the output current from the TENG was increased due to the greater pressure. As a result, the number of spikes in one touch (*N*_spike_) increased as the pressure was increased, which is a desired characteristic of the artificial mechanoreceptor module, as shown in Figure 4d.

Note that the mechanoreceptor module can respond to low pressures close to 3 kPa due to the higher output of the TENG compared to other types of nanogenerators.^[^ ^23^, ^24^ ^]^ Therefore, it can be utilized in robotics, prosthetics, and medical and healthcare devices because the human can feel gentle touches during daily activities less than 10 kPa. As mentioned earlier, *I*_in_ applied to the biristor neuron should be higher than *I*_limit_, as marked in Figure 3b. Therefore, *I*_out_ generated from the TENG should be higher than *I*_limit_ in order to generate spikes and act as an artificial mechanoreceptor. Therefore, low pressure detection is possible when the *I*_out_ generated from the TENG is larger than the *I*_limit_ of 2 nA in the biristor neuron.

The energy consumption in one spike (*E*/spike) of the artificial mechanoreceptor can be extracted with following equation:^[^ ^31^, ^32^ ^]^

(2) $$E/\mathrm{spike}=\int_{0}^{\frac{1}{f}}V_{\mathrm{out}}I_{\mathrm{in}}dt=\int_{0}^{\frac{1}{f}}V_{\mathrm{out}}I_{\mathrm{out}}dt\;$$

When the applied pressure was 3.2 kPa, the *E*/spike was extracted as 0.98 nJ per spike. Demanded energy for driving the biristor was sufficiently supplied by the TENG.

### 2.5 Pattern Recognition Simulation

Using the measured properties of the artificial mechanoreceptor module, a software simulation was conducted to classify handwritten digits using the MNIST dataset. There are two purposes of this simulation. First, we show that complex pattern recognition can be performed using the artificial mechanoreceptor module as the input neuron of a SNN. Although we show pattern recognition with handwritten digits, various object and bio‐signal patterns can be recognized for robotics, prosthetics, and medical and healthcare applications. Second, we show that the classification accuracy can be enhanced when the artificial mechanoreceptor module can respond to lower pressures, which is one of the main advantages of our device.

**Figure 5:**
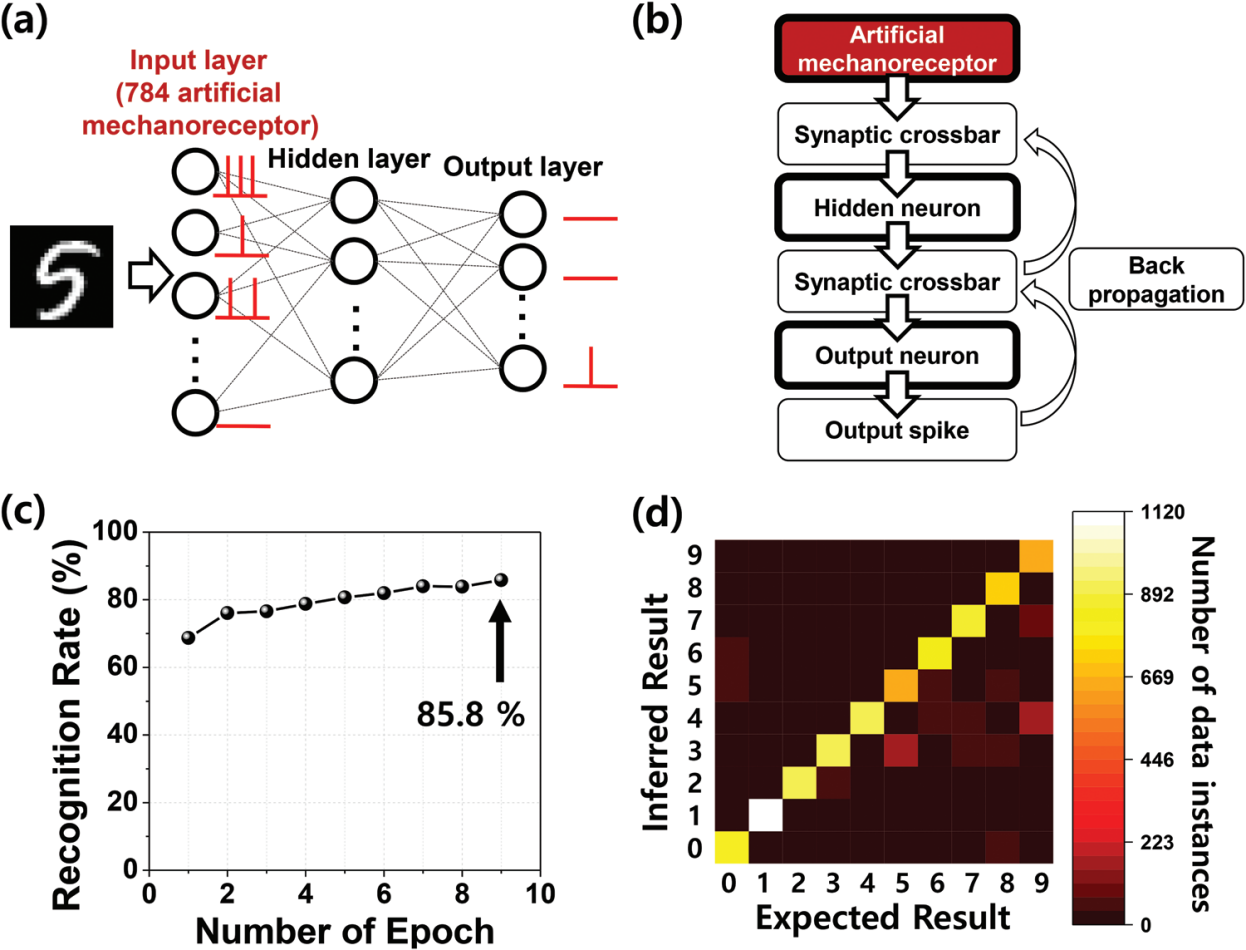
Software simulation for pattern recognition: a) Schematic of a spiking neural network (SNN) constructed for the classification of MNIST handwritten digit patterns. A three‐layer SNN composed of 784 input neurons (artificial mechanoreceptors), 100 hidden neurons, and 10 output neurons is shown. b) Flowchart of the simulation. The measured characteristics of the artificial mechanoreceptor module were reflected in the input layer. c) Recognition rate as a function of the number of epochs. Classification accuracy of 85.8% was achieved. d) Confusion matrix showing the predicted results according to the expected results. The color bar represents the number of data instances.

As shown in **Figure** **5**a, a three‐layer SNN composed of 784 input artificial mechanoreceptor modules (abbreviated as input mechanoreceptors), 100 hidden neurons, and 10 output neurons was constructed. Here, 784 input mechanoreceptors represent each pixel and 10 output neurons represent ten digits from “0” to “9.” The detailed simulation sequence is presented in Figure 5b. It should be noted that the open‐source code produced by Duan et al. was modified for our purposes.^[^ ^37^ ^]^ The measured *N*_spike_ according to the pressure was reflected in the input mechanoreceptors of the SNN. The pressure during tactile perception corresponded to the pixel intensity of the MNIST dataset. It should be noted that in addition to the measured data of *N*_spike_ according to the pressure, extra data were created by interpolation, as shown in Figure S4a, Supporting Information. In addition, virtual data that cannot respond to low pressures were created in order to confirm the degradation of the classification accuracy when the artificial mechanoreceptor module cannot respond to low pressures. The maximum applied pressure was fixed as 5 kPa because if the tactile system for the handwriting can respond to the gentle touch (<10 kPa), it can be applied to various devices including touchpads. The spikes from the input mechanoreceptors were propagated to a synaptic crossbar and weighted signals were gathered in hidden neurons. The hidden neurons were composed of IF neurons, and they generated spikes when the membrane potential reached *V*_T,firing_. The generated spikes were transmitted to the next synaptic crossbar and the weighted signals were gathered in the output neuron. Finally, the spikes of the output neurons were detected in order to predict the digits. Backpropagation was used to train the SNN by comparing predicted and expected results. Instead of a spiking neuron with a step function, a sigmoid activation function was utilized for weight updating of the synapses in order to obtain the gradient during the backpropagation of errors.^[^ ^38^ ^]^ It is worth noting that for the IF neurons composing the hidden neurons and the output neurons, *V*_T,firing_ was set to 3.7 V and *C*_par_ was set to 10 nF, reflecting the measured electrical characteristics from the fabricated biristor neuron (Figure 3c). Ideal synapses with linear and symmetric conductance changes with seven bits for their states were used.

Figure 5c shows the classification accuracy according to the training epoch when training was performed with 60 000 training data instances and validation was performed with 10 000 test data instances. Classification accuracy of 85.8% was achieved after nine epochs. Figure 5d displays a confusion matrix showing the predicted results according to the expected results for the 10 000 test data instances. The colored bar represents the number of data instances. The clear diagonal pattern shows that the handwritten digits were well classified using the artificial mechanoreceptor modules as input neurons in the SNN. It should be noted that when an artificial mechanoreceptor module cannot respond to a low‐pressure level, the classification accuracy is degraded because the information of the pixels applied via the low pressure was lost in the input layer, as shown in Figure S4b, Supporting Information. Therefore, good low‐pressure detectability of the artificial mechanoreceptor module is crucial for a high‐performance neuromorphic tactile system.

### 2.6 Hardware Implementation for Breath Monitoring

**Figure 6:**
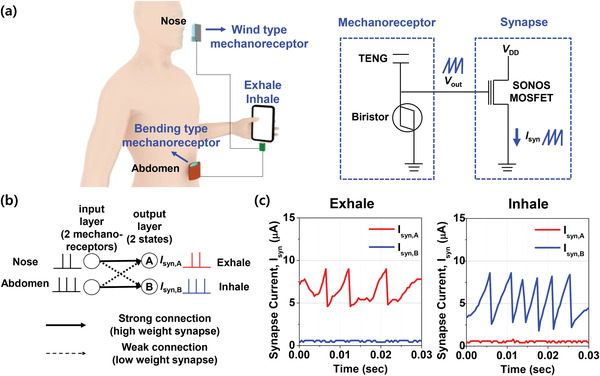
Hardware implementation for breath monitoring: a) Conceptual schematic illustration of breath monitoring for the classification of exhalation and inhalation. Two types of artificial mechanoreceptor modules were used: A wind‐type that detects wind from the nose and a bending‐type that detects movements of the abdomen. Electrical signals from the artificial mechanoreceptor were transported to SONOS‐synapses. b) Neural network based on a single‐layer spiking neural network (SNN) for the classification of breath. The input layers corresponded to the two artificial mechanoreceptor modules (wind‐type and bending‐type) and the output layers corresponded to the two states (exhale and inhale). c) Synapse current was collected at output layer A (*I*_syn,A_) and output layer B (*I*_syn,B_). Spiking was observed in output layer A for exhalation and in output layer B for inhalation.

Human breathing has been monitored as a useful indicator for real‐time health monitoring and for the early diagnosis of heart failure and sleep apnea.^[^ ^39^, ^40^ ^]^ Specifically, breath‐monitoring systems based on neural networks can be used to learn and recognize complex breathing patterns.^[^ ^41^ ^]^ We implemented a fully hardware‐based breath‐monitoring system to demonstrate further applicability of the proposed artificial mechanoreceptor module to the healthcare industry. Proposed artificial mechanoreceptor module that can respond to the small amounts of pressure is promising for the breath‐monitoring system because it can respond to exhaled wind from a nose and small movements in an abdomen. The breath‐monitoring system, capable of classifying exhalation and inhalation, was constructed based on a single‐layer perceptron (SLP). Two different types of artificial mechanoreceptor modules were used for the input neurons: A wind‐type artificial mechanoreceptor module (abbreviated as the wind‐type mechanoreceptor) that detected wind from the nose and a bending‐type artificial mechanoreceptor module (abbreviated as the bending‐type mechanoreceptor) that detected movements of the abdomen. The wind‐type mechanoreceptor mimicked the biological hair follicle receptors that detect wind pressure, while the bending‐type mechanoreceptor mimicked the biological Meissner receptor that detects low‐frequency vibrations.^[^ ^42^ ^]^ A TENG that responds to the wind pressure was designed and fabricated to construct a wind‐type mechanoreceptor. The detailed structure, operational mechanism, and output characteristics are explained in Figure S5, Supporting Information. In addition, another TENG that responds to bending pressure was designed and fabricated to construct the bending‐type mechanoreceptor, as described in Figure S6, Supporting Information. A wind‐type TENG and a bending‐type TENG were coupled with each biristor neuron in order to comprise two artificial mechanoreceptor modules. The wind‐type mechanoreceptor was positioned near the nose to detect wind from the nose and the bending‐type mechanoreceptor was positioned on the abdomen to detect the movement of the abdomen, as shown in **Figure** **6**a. The aforementioned various form factors of a TENG that can utilize numerous mechanical energy sources and respond to different types of mechanical stimuli such as wind and bending is attractive to implement diverse mechanoreceptors by the structural modification.

Referring to the circuit diagram shown in Figure 6a, electrical spike signals from the artificial mechanoreceptor modules were transported to the synapses to reflect the synaptic weight. For a synapse, a metal‐oxide‐semiconductor field‐effect transistor (MOSFET) was employed. Note that the biristor neuron does not use a gate electrode electrically even though it harnesses a physical dummy gate. Thus, the biristor neuron and the MOSFET synapse are homotypic to each other. In detail, such synaptic MOSFET encloses a charge trap layer intercalated to gate dielectrics, which has been widely utilized as a commercial flash memory cell. It is based on a SONOS structure that composes a gate poly‐crystalline Si (S), blocking SiO_2_ (O), charge trap Si_3_N_4_ (N), tunneling SiO_2_ (O), and channel single‐crystalline Si (S). Due to the structural and material homogeneity between the biristor neuron and the SONOS‐based MOSFET synapse (abbreviated as SONOS‐synapse), they can be co‐integrated. Such co‐integration of neuron devices and synaptic devices can enhance the packing density, reduce the chip cost, and simplify the fabrication procedures.^[^ ^32^ ^]^ Figure S7a,b, Supporting Information, show a SEM and a transmission electron microscope (TEM) image of the SONOS‐synapse, respectively. By controlling the electron trap density in the charge trap layer of silicon nitride (Si_3_N_4_), the threshold voltage (*V*_T_) and the conductance can be modulated to determine the synaptic weight, as shown in Figure S7c,d, Supporting Information.^[^ ^43^, ^44^ ^]^ At a fixed drain voltage of *V*_DD_ = 1 V, the output current from the SONOS‐synapse (*I*_syn_) was measured with a parameter analyzer. Although we used a parameter analyzer for this measurement, the measurement units can be commonly embedded in a chip of a mobile phone for mobile healthcare applications, as conceptually illustrated in Figure 6a.

Figure 6b shows the neural network designed for classification of exhalation and inhalation. It is composed of an input layer corresponding to two artificial mechanoreceptor modules (the wind‐type and the bending‐type) and an output layer corresponding to the two states (exhale and inhale). The wind‐type mechanoreceptor was strongly connected to output layer A, which corresponds to the exhale state, and weakly connected to output layer B, which corresponds to the inhale state. Otherwise, the bending‐type mechanoreceptor was connected strongly only to output layer B. These strong connections and weak connections are represented with the binary weights of the synapses by changing the electron trap density in the charge trap layer of the SONOS‐synapse. In other words, the high‐weight synapse for the strong connections had a low *V*_T_ and the low‐weight synapse for the weak connections had a high *V*_T_. As a result, a simple breath‐monitoring system composed of two artificial mechanoreceptor modules and four SONOS‐synapses, as depicted in the circuit diagram in Figure S8, Supporting Information, was devised. Finally, the synapse current collected at output layer A (*I*_syn,A_) and output layer B (*I*_syn,B_) was measured, as shown in Figure 6c. It was possible to classify exhalation and inhalation according to where the spike signals were detected among *I*_syn,A_ and *I*_syn,B_. In detail, a breath was classified as exhalation when spiking was observed in *I*_syn1_ and was classified as inhalation when spiking was observed in *I*_syn2_. As a result, the classification of exhalation and inhalation was accomplished using the fully hardware‐based breath‐monitoring system, which incorporated the proposed artificial mechanoreceptor modules. Although we demonstrated classification of a simple breath pattern in this work, more complex breath patterns can be classified for detecting heart failure and sleep apnea by increasing the number of hidden layers in the neural network instead of the SLP or using synapses with multiple states instead of binary states, as shown in Figure S7d, Supporting Information.

## 3 Conclusion

In this work, we proposed a self‐powered artificial mechanoreceptor module for a neuromorphic tactile system. A biological mechanoreceptor was imitated using a TENG comprising a self‐powered pressure sensor and a biristor neuron, in which spiking was varied according to the applied pressure. The proposed device can simultaneously perform pressure detection and spike encoding, which is appropriate for the input neuron of a SNN in a neuromorphic tactile system. It can greatly reduce the power consumption compared to a conventional tactile system based on the von Neumann architecture owing to its bio‐inspired neuromorphic architecture, while the artificial mechanoreceptor module itself does not use any external energy with the aid of the TENG as an energy harvester. This could not be accomplished with resistive pressure sensors or ring oscillator neurons (Table S1, Supporting Information). The mechanoreceptor module can detect low pressures near 3 kPa due to the high output range of the TENG compared to other types of nanogenerators. Using the artificial mechanoreceptor modules, classification of MNIST handwritten numbers was successfully performed (85.8%) with the aid of an experimental‐based software simulation. Furthermore, using the artificial mechanoreceptor modules and synaptic devices, a fully hardware‐based breath‐monitoring system to classify exhalation and inhalation during breathing was demonstrated. The self‐powered and low‐pressure detecting characteristics of the proposed artificial mechanoreceptor module will open a new avenue to realize tactile systems for robotics, prosthetics, and medical and healthcare applications.

## 4 Experimental Section

### Fabrication of the Triboelectric Nanogenerator

Details of the fabrication process are provided in Figure S1, Supporting Information.

### Fabrication of the Biristor Neuron

A biristor neuron with a base width (*W*_B_) of 250 nm and base length (*L*_B_) of 500 nm was fabricated on a SOI wafer. Figure S2, Supporting Information, presents the details of the fabrication process.

### Measurement Setup

The setup to measure the characteristics of the artificial mechanoreceptor module consists of a vibration generating part and an electrical measurement part. For the vibration generation part, a function generator (33 120, hp) was used to generate and transport an electric signal to a power amplifier (pa‐141, Labworks) connected to an electrodynamic shaker (LW‐140‐110, Labworks). For the electrical measurement part, a semiconductor parameter analyzer (B1500A, Keysight) was used. To measure the electrical properties of only the TENG, an electrometer (6514, Keithley) was used. To measure the electrical properties of only the biristor neuron and the SONOS‐based MOSFET synapse, a semiconductor parameter analyzer (B1500A, Keysight) was used.

### Operation of the Wind‐Force Detecting Triboelectric Nanogenerator

The detailed structure, operational mechanism, and output characteristics are presented in Figure S5, Supporting Information.

### Operation of the Bending‐Force Detecting Triboelectric Nanogenerator

The detailed structure, operational mechanism, and output characteristics are presented in Figure S6, Supporting Information.

### Scanning Electron Microscope Analysis

SEM images were taken using a Magellan 400 field‐emission SEM (FEI Company).

### Transmission Electron Microscope Analysis

TEM images were taken using a FE‐STEM (HD‐2300A) device made by Hitachi High‐Technologies Corporation.

### Software‐Based Simulation

Software simulations for classification of handwritten digits in the MNIST dataset were performed using Python. The measured number of spikes during one touch (*N*_spike_) according to the pressure of an artificial mechanoreceptor module was reflected in the simulation.

## Conflict of Interest

The authors declare no conflict of interest.

## Supporting information

Supporting Information

## Data Availability

The data that support the findings of this study are available from the corresponding author upon reasonable request.
