# Self‐Powered Artificial Mechanoreceptor Based on Triboelectrification for a Neuromorphic Tactile System

**DOI:** 10.1002/advs.202201831

**Published:** 2022-05-05

**Authors:** Joon‐Kyu Han, Il‐Woong Tcho, Seung‐Bae Jeon, Ji‐Man Yu, Weon‐Guk Kim, Yang‐Kyu Choi

*Adv. Sci*. **2022**, *9*, 2105076

DOI: 10.1002/advs.202105076

In the originally published version of article, the affiliation of Seung‐Bae Jeon is incorrect. Please find the correct affiliations here:

J.‐K. Han, I.‐W. Tcho, J.‐M. Yu, W.‐G. Kim, Y.‐K. Choi

School of Electrical Engineering

Korea Advanced Institute of Science and Technology (KAIST)

291 Daehak‐ro, Yuseong‐gu, Daejeon 34141, Republic of Korea

E‐mail: ykchoi@ee.kaist.ac.kr

S.‐B. Jeon

Department of Electronic Engineering

Hanbat National University

125 Dongseo‐daero, Yuseong‐gu, Daejeon 34158, Republic of Korea

