# Supplementary material for: Self‐Powered Artificial Mechanoreceptor Based on Triboelectrification for a Neuromorphic Tactile System
Source: Adv Sci (Weinh). 2022 Jan 14;9(9):2105076. doi: 10.1002/advs.202105076 (PMC8948587; doi:10.1002/advs.202105076)
Supplement: Supplementary file 1 — Supporting Information [file ADVS-9-2105076-s001.pdf]

ADVANCED SCIENCE
Open Access

Supporting Information

for *Adv. Sci.*, DOI 10.1002/advs.202105076

Self-Powered Artificial Mechanoreceptor Based on Triboelectrification for a Neuromorphic Tactile System

*Joon-Kyu Han, Il-Woong Tcho, Seung-Bae Jeon, Ji-Man Yu, Weon-Guk Kim and Yang-Kyu Choi**

© 2020 Wiley-VCH GmbH

ADVANCED SCIENCE
Open Access

Supporting Information

for *Adv. Sci.*, DOI: 10.1002/advs.202105076

Self-powered Artificial Mechanoreceptor based on Triboelectrification for a Neuromorphic Tactile System

*Joon-Kyu Han, Il-Woong Tcho, Seung-Bae Jeon, Ji-Man Yu, Weon-Guk Kim, and Yang-Kyu Choi**

Supporting Information

Self-powered Artificial Mechanoreceptor based on Triboelectrification for a Neuromorphic Tactile System

Joon-Kyu Han[†], *Il-Woong Tcho*[†], *Seung-Bae Jeon, Ji-Man Yu, Weon-Guk Kim, and Yang-Kyu Choi**

[†]These authors equally contributed to this work.

Supplementary figures

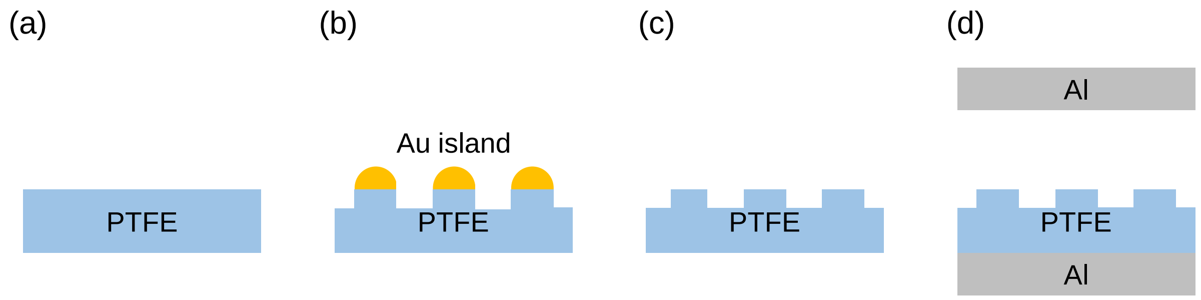

Figure S1. Fabrication procedure of the TENG: a) PTFE film with a thickness of 100 μm was selected as a triboelectric material. b) Au was evaporated on the PTFE film with a targeted thickness of 4 nm. With this low thickness, island-like Au nanoparticles were formed. c) The PTFE film was etched by plasma with a gas mixture ($Ar{:}O_2{:}CF_4$ = 15:10:40). d) Two Al plates were selected as electrodes for the TENG. One Al plate was used as the top electrode and the other Al plate attached on the rear surface of the PTFE was used as the bottom electrode.

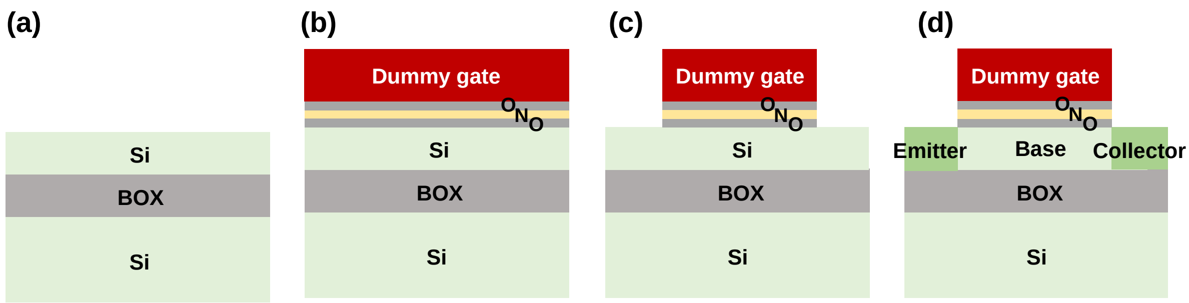

Figure S2. Fabrication procedure of the biristor: a) A p-type (100) SOI wafer with a buried oxide (BOX) thickness of 140 nm was selected as the starting wafer. The Si was thinned down to 50 nm, and the active area was patterned using photo-lithography and plasma etching. b) O/N/O and n^+ poly-Si were sequentially deposited for dummy dielectrics and a gate. c) The dummy gate was patterned by photo-lithography and plasma etching. d) Emitter/collector doping was performed by arsenic implantation and subsequent rapid thermal annealing (RTA). Base doping was performed by boron implantation and subsequent RTA. After the fabrication, the biristor can be transferred onto various flexible substrates through wafer transfer technology. It is attractive to transfer an artificial mechanoreceptor for robotics, medical and healthcare devices.

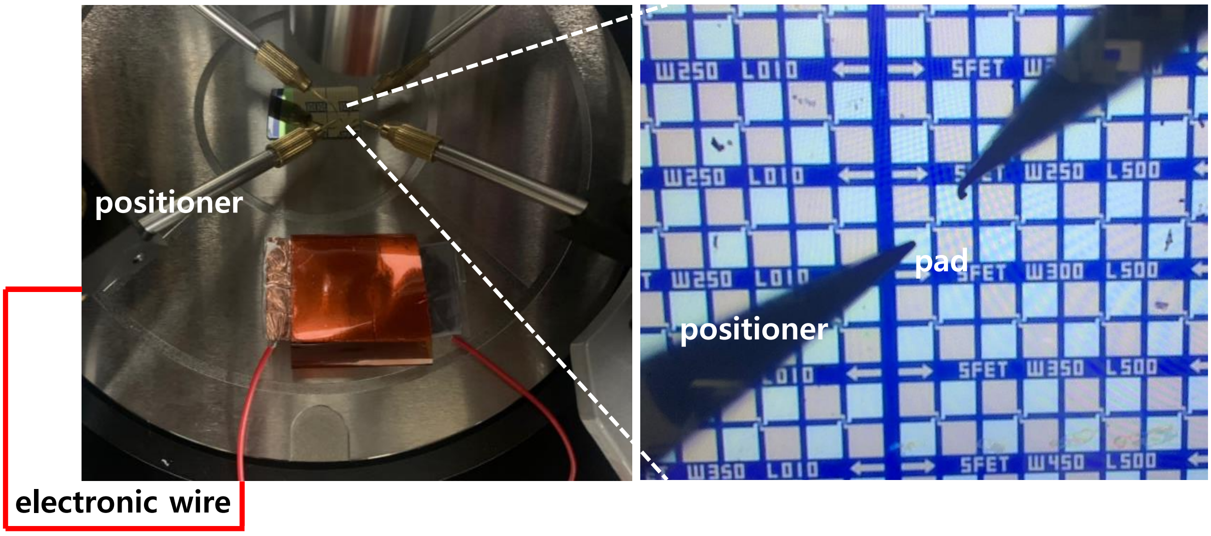

Figure S3. The connection of the TENG and the biristor neuron. The TENG and the biristor neuron were electrically connected using a probe positioner and an electronic wire. The red line outside the left figure is illustrated for electrical connection between the probe positioner and the TENG.

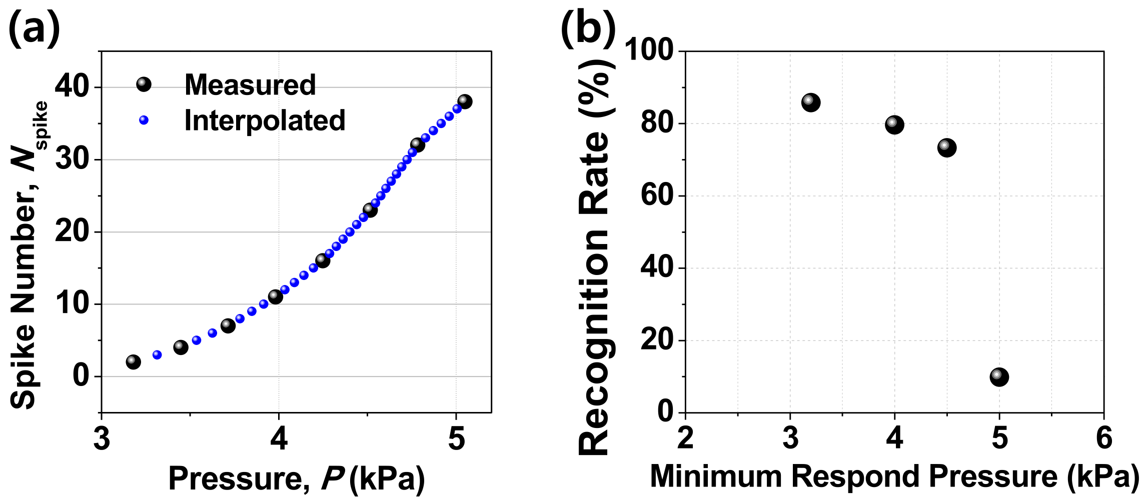

Figure S4. Detailed classification of handwritten digits in the MNIST dataset: a) The measured number of spikes during one touch (N_{spike}) according to the pressure with their interpolated N_{spike} for the simulation. In addition to the measured data, extra data were created by interpolation. b) The recognition rate depending on the minimum response pressure. The recognition rate was degraded when the artificial mechanoreceptor could not respond to lower pressure levels.

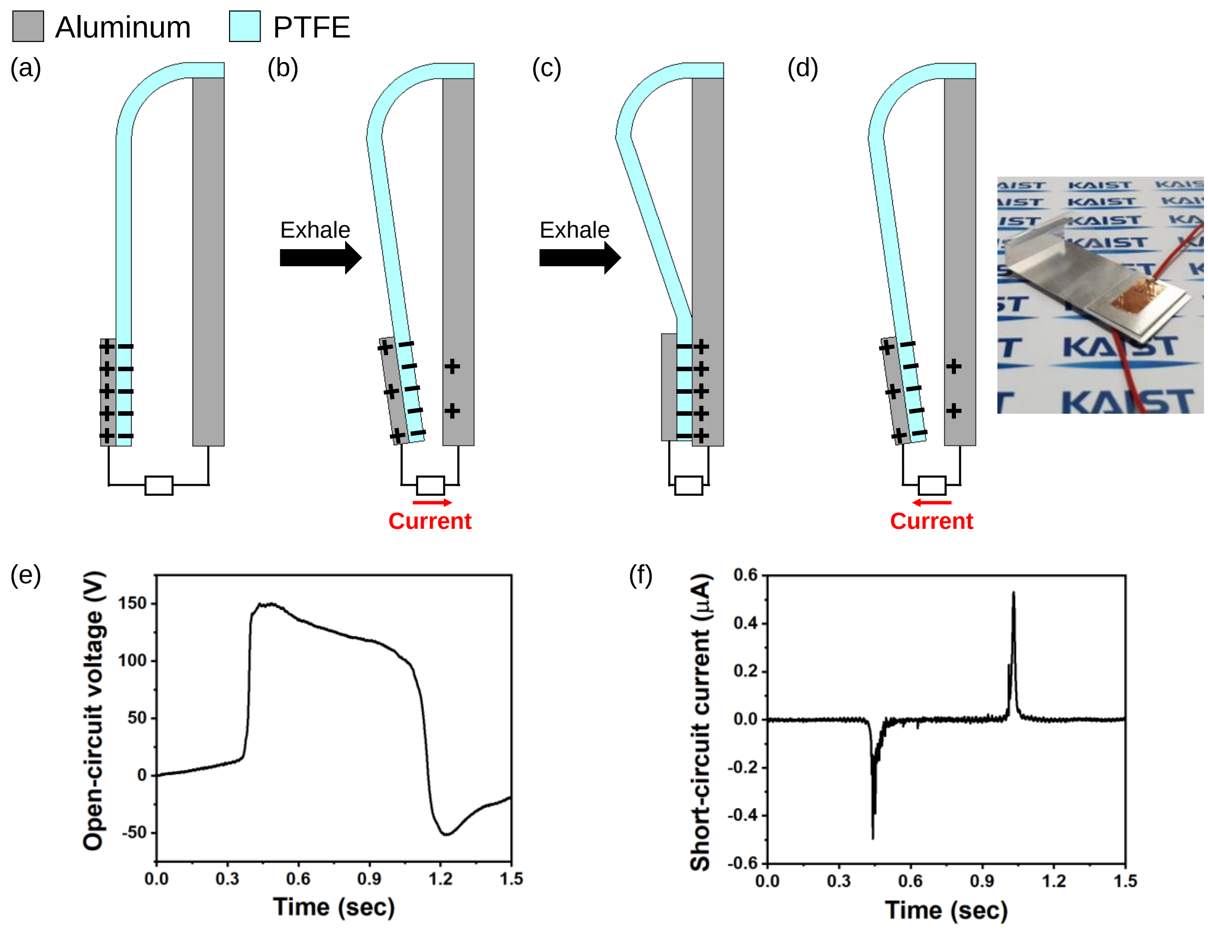

Figure S5. Operational mechanism and output characteristics of the TENG to detect wind pressure. The fabricated wind-type TENG consists of a PTFE film layer and two Al electrodes. The electrode on the left is attached to the rear surface of the PTFE film and the electrode on the right is an Al plate. a) The surface of the PFA film is charged negatively *via* contact electrification with the Al plate. b) When wind pressure is applied to the PTFE film, the film moves close to the Al plate. At this point, current flows from the electrode on the left to the electrode on the right. c) When the PTFE film is in complete contact with the Al plate, the current stops flowing. d) Without wind pressure, the PTFE film moves away from the Al plate. At this point, current flows from the electrode on the right to the electrode on the left. The inset image shows an optical photograph of the fabricated wind-type TENG. e) Open-circuit voltage of the wind-type TENG operated by actual exhalation. f) Short-circuit current of the wind-type TENG operated by actual exhalation.

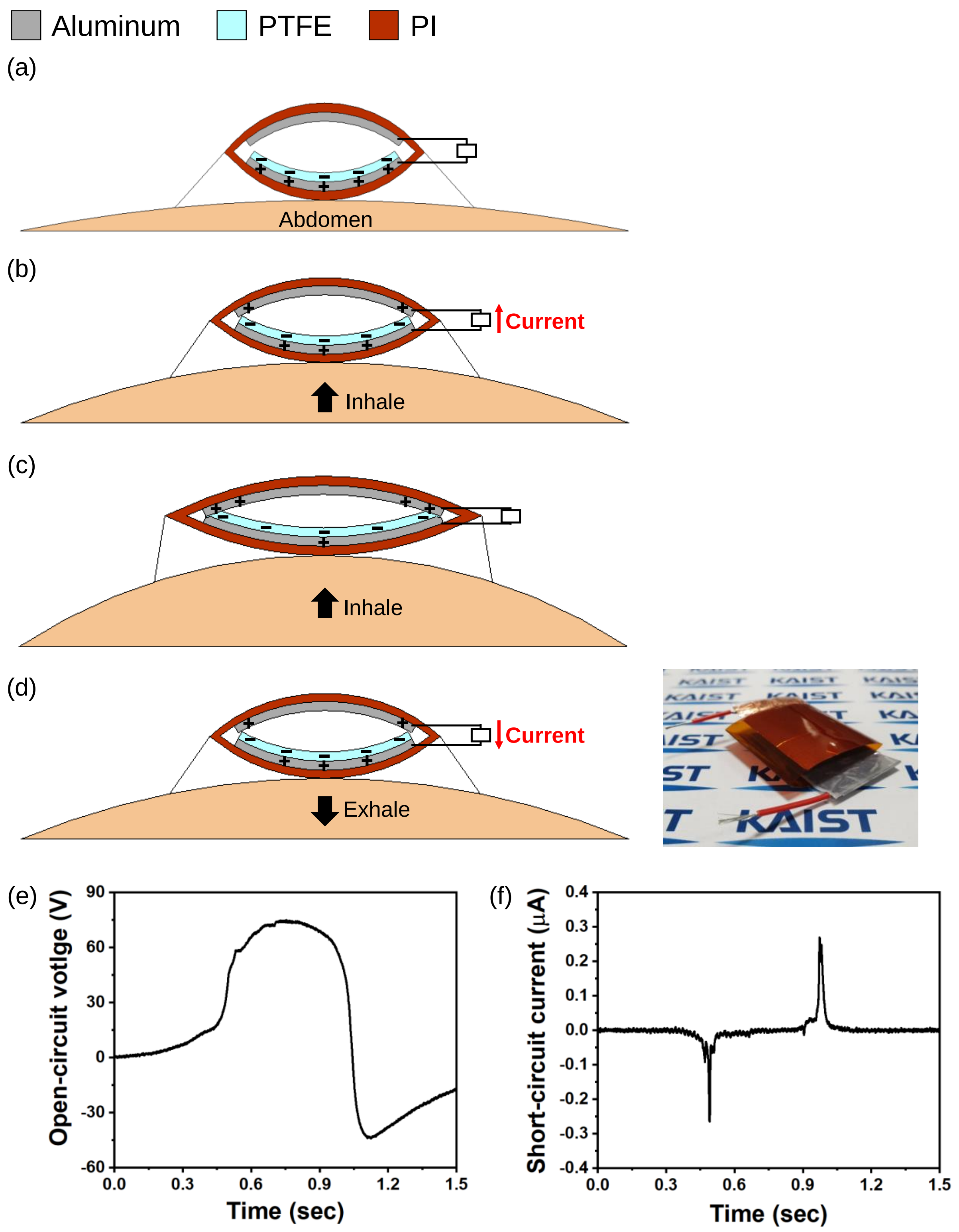

Figure S6. Operational mechanism and output characteristics of the TENG to detect bending pressure. The fabricated bending-type TENG consists of two thick arch-shaped polyimide (PI) films that serve as a skeleton. On the inner surface of the upper PI film, an Al electrode is attached. On the inner surface of the PI film, an Al electrode and PTFE film are attached. This bending-type TENG

attached on the abdomen of a human can detect inhalation movements. a) The surface of the PTFE film is charged negatively *via* contact electrification with the Al plate. b) When the abdomen swells by inhalation and bending force is applied to the TENG, the PTFE film moves closer to the Al plate. At this point, current flows from the lower electrode to the upper electrode. c) When the swelling of the abdomen stops, the current stops flowing. d) During exhalation, the PTFE film moves away from the Al plate. At this point, current flows from the upper electrode to the lower electrode. The inset image shows an optical photograph of the fabricated bending-type TENG. e) Open-circuit voltage of the bending-type TENG operated by actual inhalation. f) Short-circuit current of the bending-type TENG operated by actual inhalation.

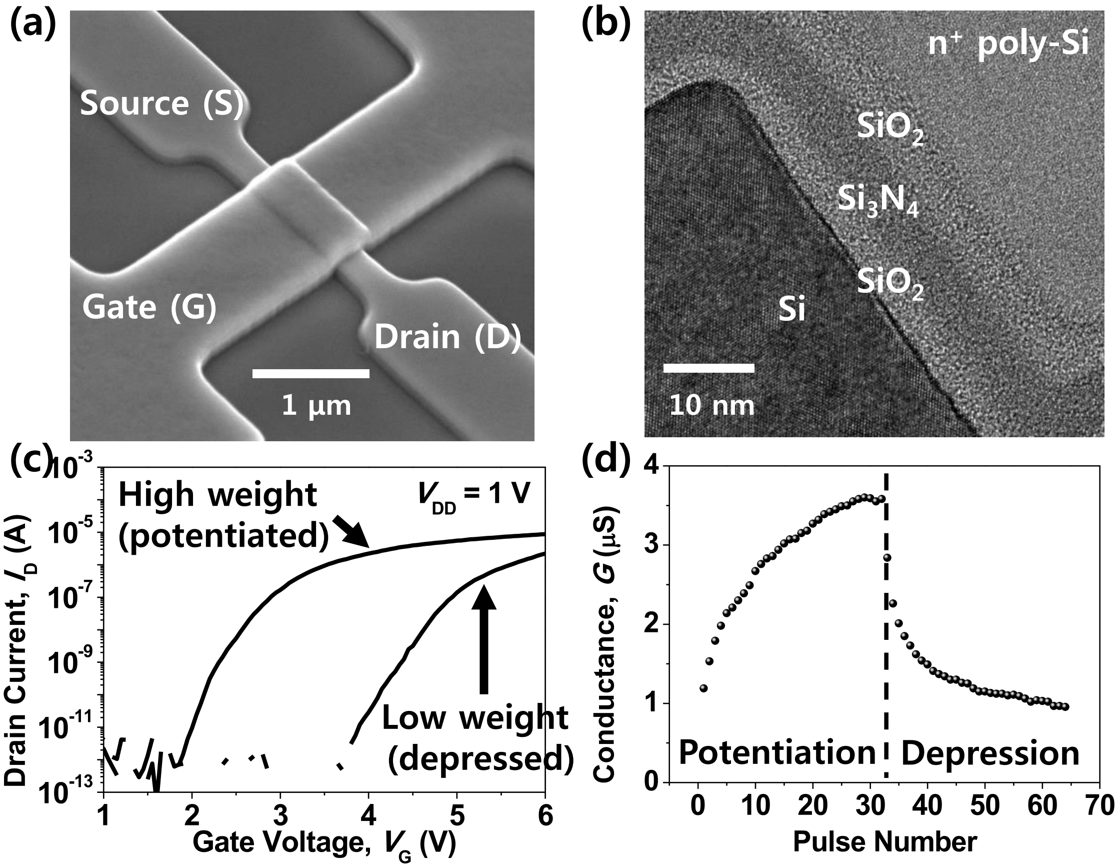

Figure S7. Silicon-oxide-nitride-oxide-silicon (SONOS) MOSFET synapse (SONOS-synapse): a) Scanning electron microscope (SEM) image and b) transmission electron image (TEM) of the SONOS-synapse.

The electron density in the charge trap layer of silicon nitride (Si_3N_4) can modulate the threshold voltage (V_T) and the conductance, determining the synaptic weight. c) Transfer characteristics (I_D-V_G) of the SONOS-synapse with a high weight (potentiated) and a low weight (depressed). V_T shifted leftward after potentiation, showing a higher synaptic weight, and shifted rightward after depression, showing a lower synaptic weight. A gate voltage with a pulse magnitude of 13 V and a pulse width of 1 ms was applied to create the low weight state. d) Potentiation-depression (P-D) characteristics of the SONOS-synapse to show conductance modulation of multi-states (5 bits; 2^5=32 states). Voltage with a pulse magnitude of -10 V and a pulse width of 10 ms was applied to the gate for potentiation, while voltage with a pulse magnitude of 9 V and a pulse width of 1 µs was applied to the gate for depression. The reading gate voltage was set to 4 V.

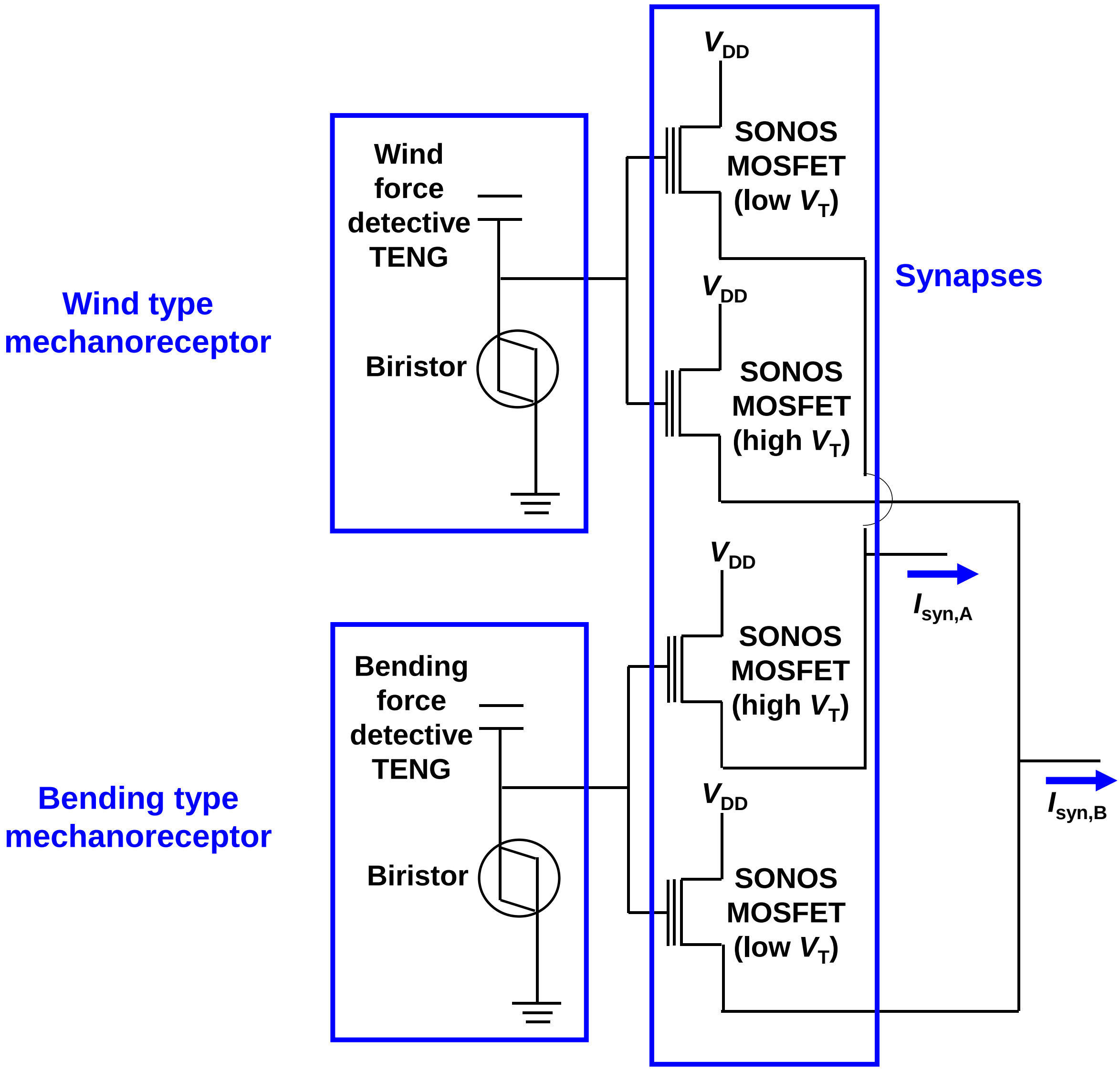

Figure S8. Circuit diagram of the breath-monitoring system. A breath-monitoring system composed of two artificial mechanoreceptor modules and four SONOS-synapses can classify exhalation and inhalation. Drain voltage applied to the SONOS-synapses (V_{DD}) was set to 1 V.

Ref.	Architecture	Pressure range	Self-powered	Software implementation	Hardware implementation
**[S1]**	Organic resistive pressure sensor + organic ring oscillator	> 2.5 kPa	No	Not shown	Reflex arc
**[S2]**	Piezoelectric generator + Mott memristor	~100 kPa	Yes	Not shown	Not shown
**[S3]**	Resistive pressure sensor + Mott memristor	> 0.4 kPa	No	Pulse coupled neural network	Multisensory tactile integration
**This work**	**TENG + biristor**	**> 3.2 kPa**	**Yes**	**Classification of MNIST hand-written digits**	**Breath monitoring**

Table S1. Comparison with previously reported artificial mechanoreceptors that can detect pressure and generate spike signals. Due to the self-powered ability with high output of the TENG, a self-powered artificial mechanoreceptor module capable of responding to low-pressure levels due to high output from the TENG was implemented for the first time. Both software-based classification of handwritten digits in the MNIST dataset and fully hardware-based breath monitoring were also demonstrated for the first time.

References

[S1] Y. Kim, A. Chortos, W. Xu, Y. Liu, J. Y. Oh, D. Son, J. Kang, A. M.. Foudeh, C. Zhu, Y. Lee, S. Niu, J. Liu, R. Pfattner, Z. Bao, T. W. Lee, *Science* **2018**, *360*, 998.

[S2] X. Zhang, Y. Zhuo, Q. Luo, Z. Wu, R. Midya, Z. Wang, W. Song, R. Wang, N. K. Upadhyay, Y. Fang, F. Kiani, M. Rao, Y. Yang, Q. Xia, Q. Liu, M. Liu, J. J. Yang, *Nat. Commun*. **2020**, *11*, 51.

[S3] F. Li, R. Wang, C. Song, M. Zhao, H. Ren, S. Wang, K. Liang, D. Li, X. Ma, B. Zhu, H. Wang, Y. Hao, *ACS Nano* **2021**, in press.
